# Prediction of Hypertension Outcomes Based on Gain Sequence Forward Tabu Search Feature Selection and XGBoost

**DOI:** 10.3390/diagnostics11050792

**Published:** 2021-04-27

**Authors:** Wenbing Chang, Xinpeng Ji, Yiyong Xiao, Yue Zhang, Bang Chen, Houxiang Liu, Shenghan Zhou

**Affiliations:** School of Reliability and Systems Engineering, Beihang University, Beijing 100191, China; changwenbing@buaa.edu.cn (W.C.); sy1914103@buaa.edu.cn (X.J.); xiaoyiyong@buaa.edu.cn (Y.X.); Zhangyue1127@buaa.edu.cn (Y.Z.); bang@buaa.edu.cn (B.C.); zy1914125@buaa.edu.cn (H.L.)

**Keywords:** hypertension outcomes, biomedical engineering, feature selection, gain sequence forward tabu search, disease prediction, XGBoost

## Abstract

For patients with hypertension, serious complications, such as myocardial infarction, a common cause of heart failure, occurs in the late stage of hypertension. Hypertension outcomes can lead to complications, including death. Hypertension outcomes threaten patients’ lives and need to be predicted. In our research, we reviewed the hypertension medical data from a tertiary-grade A class hospital in Beijing, and established a hypertension outcome prediction model with the machine learning theory. We first proposed a gain sequence forward tabu search feature selection (GSFTS-FS) method, which can search the optimal combination of medical variables that affect hypertension outcomes. Based on this, the XGBoost algorithm established a prediction model because of its good stability. We verified the proposed method by comparing other commonly used models in similar works. The proposed GSFTS-FS improved the performance by about 10%. The proposed prediction method has the best performance and its AUC value, accuracy, F1 value, and recall of 10-fold cross-validation were 0.96. 0.95, 0.88, and 0.82, respectively. It also performed well on test datasets with 0.92, 0.94, 0.87, and 0.80 for AUC, accuracy, F1, and recall, respectively. Therefore, the XGBoost with GSFTS-FS can accurately and effectively predict the occurrence of outcomes for patients with hypertension, and can provide guidance for doctors in clinical diagnoses and medical decision-making.

## 1. Introduction

Hypertension outcomes can include serious complications (e.g., cerebrovascular disease, myocardial infarction, stroke, etc.), and even death, when the condition progresses to a terminal stage. Hypertension is one of the most common chronic human diseases, and serious hypertension complications can greatly endanger the life and health of a patient, causing irreversible damage to the patient’s heart, brain, kidney, and fundus.

Heart complications of hypertension mainly include left ventricular hypertrophy, angina pectoris, myocardial infarction, and heart failure. Hypertension can damage the heart blood vessels, mainly the coronary arteries, which will eventually cause atherosclerosis of the coronary arteries. The myocardial blood supply is reduced, causing coronary heart disease. The brain complications of hypertension mainly include hemorrhagic stroke, ischemic stroke, hypertensive encephalopathy, etc. Among them, cerebral hemorrhage is one of the most serious hypertension complications. The kidney complications of hypertension mainly include malignant arterioles nephrosclerosis and chronic renal failure. The main manifestations of hypertension on the kidneys are proteinuria and impaired renal function. Some patients tend to have the impaired distal renal tubular concentration in the late stage of hypertension. Fundus complications of hypertension include retinal arteriosclerosis. Patients may experience decreased vision, bleeding in the fundus, cataracts, and blindness.

Due to the severe outcomes, patients and doctors are aiming to prevent the occurrence and progression of hypertension. To effectively reduce the incidence of outcomes, people must first effectively predict the outcomes and prevent them before they occur. However, it is still very difficult to detect the threat of outcomes because there are no obvious early signs of hypertensive complications. In addition, there are few studies on hypertension outcome predictions; the current research is mainly from a medical perspective and it is hard to predict.

Computer science and data analysis technology provide new methods and ideas for the prediction of hypertension outcomes. On the one hand, machine learning, data mining, and information sciences have been widely used in various fields of medicine and achieved good results [1,2,3,4,5,6,7], providing technical support for this study. On the other hand, the application of electronic medical records and databases, automatic and electronic medical equipment, and increasing emphasis placed on medical data by hospitals and medical institutions all promote the digitalization of medical information. As a result, massive amounts of medical data on hypertension patients were obtained and preserved, providing data for this study.

This paper reviews the medical data of hypertension patients provided by the hypertension center of a tertiary-grade A class hospital in Beijing as the research object, and accurately predicts the occurrence of outcomes by machine learning technology. The purpose of this study was to map the relationships between medical indicators and outcomes through analyses of hypertension medical data. Therefore, when the medical data of a new patient was input, the model determined whether the hypertension outcomes occurred with a certain probability. This was a supervised machine learning classification problem with two main tasks: (1) to reduce the dimension of medical data characteristics. The patients’ medical data indices were numerous, with high dimensions, including blood pressure index, blood routine, urine, routine, etc. From the perspective of data mining, the high dimension of the data set had irrelevant, redundant information and noise, which affected the accuracy of the prediction model. From the perspective of a medical application, the less indicators needed to predict the outcomes, the less difficult the indicator acquisition, and the lower the prediction cost. (2) The second task was to establish a machine learning model to predict hypertension outcomes and evaluate the predictive performance of the model.

In the first task, this paper constructed the feature selection method based on the gain sequence forward tabu search (GSFTS) to automatically select the high-quality feature combination. This method can greatly reduce the data dimension and improve the prediction accuracy. More importantly, the feature selection method automatically helps doctors identify the key factors for hypertension outcomes in a large number of medical indicators. In the second task, this paper adopted XGBoost to realize the prediction of hypertension outcomes.

Feature selection is the process of selecting a feature subset in a given set of attributes. The dimensions of medical data are usually very high. It is important to select the best feature subset to reduce the processing cost, and improve the practicability of the model constructed from it.

Search strategy and feature evaluation functions are the key steps of feature selection. The wrapper method uses the classifier performance as the evaluation function of feature selection. The embedded method combines the process of feature selection with the process of learning. The filter feature selection method first carries on the feature selection before training the learner [8,9]. Sequence search strategy refers to adding or deleting one or more features in each step, and the feature evaluation function is used to determine whether the deletion or addition is effective.

Therefore, feature selection is essentially a search optimization problem, and heuristic algorithms, such as genetic algorithm (GA), simulated annealing algorithm (SA), ant colony algorithm (ACA), and tabu search (TS), are a few options to solve the combination optimization problem and find better solutions. Heuristic algorithms were widely used in feature selection and have achieved good results in medicine [10,11].

XGBoost (Extreme Gradient Boosting) is a commonly used and efficient algorithm for machine learning, and its effect is remarkable [12,13,14,15,16]. For example, CYe (2018) et al. constructed a risk prediction model for essential hypertension using electronic health data and XGBoost algorithm [13]. Y Liu compared four machine learning algorithms through experiments and proved that XGBoost has advantages in predicting hypertension outcomes [16].

At present, some scholars have applied machine learning methods to the prediction of hypertension complications and other related diseases [17,18,19,20,21,22]. The methods include random forests, support vector machines, logistic regression, decision trees, etc. We noticed that these studies focus on the use of traditional and standard machine learning algorithms to build models, without considering the impact of medical features on the prediction results, and the efficiency of machine learning under the condition of large-scale patient data. Therefore, on the one hand, we proposed a new feature selection method to better explore what medical features could affect the hypertension outcomes. On the other hand, we used a novel integrated learning method XGBoost to efficiently process large-scale medical data and meet actual needs. Therefore, the method proposed in this study is innovate, integrating theory and practice.

This article is divided into four parts. The specific organizational structure is as follows: Section 1 mainly introduces the research background, literature review, research contents, and ideas. Section 2 introduces the method and model, and proposes a hypertension outcome prediction model based on GSFTS-FS and XGBoost. Section 3 is an empirical study, the results of which are analyzed and discussed to verify the effectiveness of the proposed method. Section 4 is the conclusion.

## 2. Materials and Methods

### 2.1. Medical Data and Preprocessing

The medical data used in this study were obtained from a hypertension center of a tertiary-grade A class hospital in Beijing. The hospital collected data from 1357 patients with hypertension from September 2012 to December 2016. The patients come from various regions in China. The data set were divided into two parts. One part was the medical examination data and related survey data (i.e., characteristic data) during the patient admission. The other part involved the data on whether the outcomes occurred in the patients (i.e., the labeled data: yes/no or 1/0) marked by the hospital staff during the follow-up period after the patient was discharged. Characteristic data included baseline data, limb blood pressure, ambulatory blood pressure, echocardiography, heart failure, and other categories—a total of 132 examination indicators. The outcomes involved complications of the four target organs: heart, brain, kidney, and fundus. Table 1 shows the name, medical description, data type, mean value, standard deviation, and data distribution range of some medical indicators of the data set. Table A1 in Appendix A is a list of all the medical features that are considered in this study.

There are impurities in the original data. (1) There are a large number of missing values in the original data set, and some attributes have missing values of more than 90%. (2) There are some abnormal values, which exceed the regular distribution interval of the attribute. (3) For different physical examination indicators, the attribute dimensional units are different.

The deletion and mean interpolation are used to deal with missing values. Features and samples with missing values exceeding 50% are directly deleted; for deleted data sets, missing values are interpolated according to the mean value of attributes. Outliers are directly deleted. In this study, the maximum and minimum standardization methods are used to unify the dimensions.

### 2.2. Gain Sequence Forward Tabu Search Feature Selection (GSFTS-FS)

**Figure 1:**
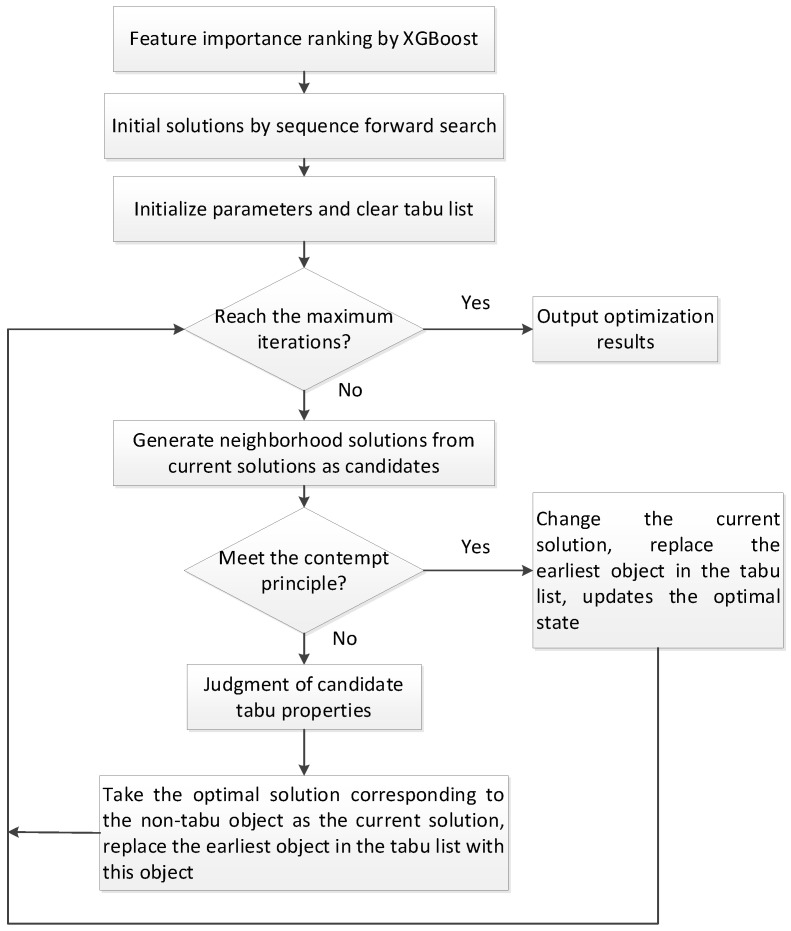
Basic steps of the gain sequence forward tabu search (GSFTS) algorithm.

In this study, we proposed a new medical feature selection (FS) strategy called gain sequence forward tabu search (GSFTS). GSFTS-FS is a wrapper feature selection method. It takes the performance of the prediction model as a criterion and objective function to evaluate the quality of the selected feature subset. It is mainly divided into three steps. First, XGBoost rank and score feature importance based on the average gain. Second, sequence forward search based on the ranking is performed to obtain initial feature combinations. Finally, the selected feature combination is further optimized by tabu search algorithm. The basic steps of the GSFTS-FS algorithm are shown in Figure 1.

Based on the concept of the GSFTS algorithm, the specific process is as follows:

1. Feature importance ranking.

We first build an initial classifier (XGBoost) and fit the data. We calculate the average information gain across all split points in XGBoost of each feature to rank all feature importance. The higher the gain, the greater the feature contribution and the higher the importance.

2. Initial Solution by gain-based sequence forward search.

The traditional tabu search algorithm has two shortcomings: (1) Strong dependence on the initial solution. A good initial solution helps the search to reach the optimal solution quickly, while a bad initial solution often makes the search difficult or impossible to reach the optimal solution. (2) The running time of the algorithm is greatly affected by the initial solution. A better initial solution can push the search move closer to the optimal solution with fewer iterations, thereby reducing the search time. The search with poor initial solution needs many iterations to get close to the optimal solution, which prolongs the search time.

Aiming to provide a better initial optimal solution for tabu search, we proposed a new Sequence Forward Search based on feature importance ranking by information Gain (gain-based SFS). Suppose the feature importance is ranked as (Fa, Fb, Fc…), specific steps are as follows:

(1) Add the feature Fa, which ranks first in importance, to the feature subset *S*. The current subset is *S*′ = {Fa}, the dimension of the subset is *i* = 1, and the classification accuracy on the training set is selected as the evaluation function *f*.

(2) Calculate the evaluation function score under the current feature subset *f*(*S*′).

(3) According to the order of feature importance, the feature with ranking *i* + 1 is added to feature subset *S*′.

(4) Calculate the evaluation function score *f*(*S*′) under the current feature subset, if the score drops, stop searching; if the score rises, repeat step (3).

3. Encoding.

**Figure 2:**
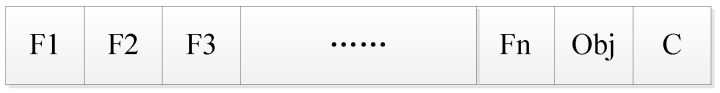
Code structure.

**Figure 3:**
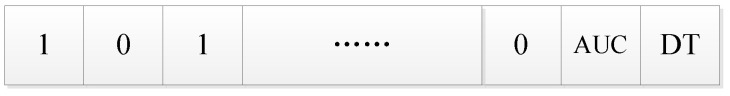
Initial solution example by SFS after encoding.

We propose a coding structure as shown in Figure 2 before tabu search. It consists of three parts. The first part F1, F2, F3… Fn represents each feature in an n-dimensional medical feature set by a 0/1-bit string. If the feature is in the feature subset, then Fi (i ∈ [1, n]) is 1, or else it is 0. The second part is the objective function (accuracy, precision, recall, F1, and AUC), and the third part is the selected classification algorithm. Figure 3 is an example of an initial solution after encoding.

4. Neighborhood feasible solution.

**Figure 4:**
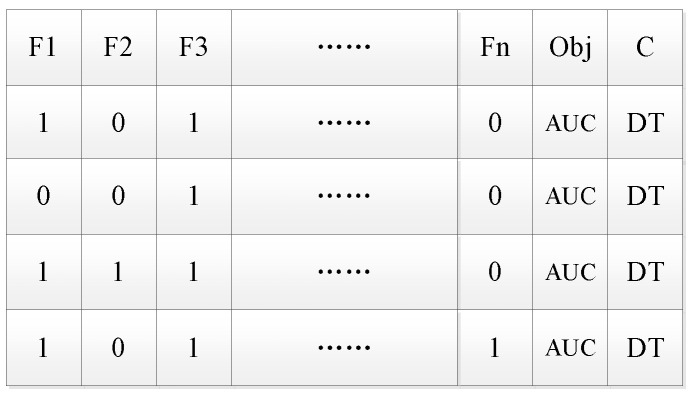
An example of the neighborhood feasible solutions.

It is important to generate a neighborhood feasible solution based on the current solution. The specific method is to randomly select the feature code in the initial solution. If the feature number is 0, add the feature (the code is changed to 1); if the feature number is 1, then delete the feature (the code is changed to 0). Each neighborhood feasible solution differs from the initial solution by only one feature code. Then a specified number of neighborhood feasible solutions are generated. The number of feasible solutions in the neighborhood is the candidate set length. Figure 4 shows the four neighborhood feasible solutions generated from the initial solution.

According to selected classifier DT and evaluation function AUC, the optimal solution of the four feasible solutions in the neighborhood is selected and regarded as the current optimal solution in the next iteration.

5. Tabu movement.

If the feature i (added or deleted) makes the neighborhood feasible solution the current optimal solution, then the feature i cannot be selected (added or deleted) in the next several rounds of T (tabu list length) iterations. For example, the third feasible solution in Figure 4 becomes the current optimal solution because the feature F2 is added to the initial solution. Then feature F2 is added to the tabu list. Table 2 is a tabu list with tabu length TL = 3. In the next 3 iterations, F2 cannot be added or deleted. The tabu list guarantees that the algorithm prevents searching for solutions that have been accessed, and helps to jump out of local optimal solutions.

6. Contempt principle.

Due to the existence of the tabu list, generally tabu feature will not participate in the next several rounds of search. However, when the participation of the tabu feature can make the evaluation function reach the historical optimal, the tabu feature will be amnesty, which is conducive to finding the global optimal solution. Specifically, if moving (adding/deleting) feature i can make the feasible solution better than any solution of the previous iteration, then it is allowed to add/delete the feature, even if feature i is in the tabu list. For example, if moving feature F2 in Table 2 in the next three rounds of iteration can make the feasible solution the historically optimal solution, then remove F2 from the tabu list. The contempt principle is a method of covering tabu movement, which can avoid missing a good solution.

7. Stop rule.

We set the stop rule to a fixed number of iterations.

In summary, GSFTS-FS has the following advantages:

In gain-based-SFS, the order of adding features is arranged in order of feature importance. The more important features are prioritized and added to the feature combination until the classification algorithm reaches a certain local optimal solution. Therefore, medical features that have a significant impact on the hypertension outcomes are given priority to provide a good initial solution for the subsequent tabu search.

The tabu search optimizes the gain-based-SFS solution, which push the algorithm jump out of the local optimal solution and continue the search. The tabu search uses a tabu list to record the local optimal points that have been reached. In the next search, the information in the tabu list is used to no longer or selectively search for these points, so as to avoids converging into a local optimum.

### 2.3. XGBoost Model for Hypertension Outcomes Prediction

#### 2.3.1. Model Mathematical Theory

XGBoost is an ensemble learning algorithm, and it is one of the boosting algorithms. The idea of XGBoost is to continuously add trees, and continuously perform feature splitting to grow a tree. Each time a tree is added, it is actually learning a new function to fit the residuals of the last prediction. When k trees are obtained after training, the score of a sample is predicted. In fact, according to the characteristics of this sample, a corresponding leaf node will fall in each tree, and each leaf node corresponds to a score. The scores corresponding to each tree add up to the predicted value of the sample.

XGBoost uses the second-order Taylor expansion of the loss function and adds a regular term to balance the complexity of the model and the decline in the loss function. It seeks the best solution globally and well avoids model overfitting. Suppose that the model generates *t* decision trees. Its prediction value for sample *i* is as follows.

(1) $${\overset{\wedge }{y}}_{i}^{\left(t\right)}=\sum_{k=1}^{t}f_{k}(x_{i})={\overset{\wedge }{y}}_{i}^{\left(t-1\right)}+f_{t}(x_{i}),f_{k}\in F,i\in n,$$

${\overset{\wedge }{y}}_{i}^{\left(t\right)}$ represents the predicted value of sample i, which is based on the sum of the predicted values of *t* decision trees. *n* represents the total number of all samples, and the subscript i represents the *i*-th sample. $f_{t}$ is the *t*-th classification tree, and *F* is the set space of all trees.

The loss function is as follows:

(2) $$L^{\left(t\right)}=\sum_{i=1}^{n}l(y_{i},{\overset{\wedge }{y}}_{i}^{\left(t\right)})+\sum_{k=1}^{t}\Omega (f_{k})$$

l represents the degree of deviation between the predicted value ${\overset{\wedge }{y}}_{i}^{\left(t\right)}$ and the true value $y_{i}$; the second half of formula (2) represents the sum of the complexity of each tree and $\Omega (f_{k})=\gamma *T+1/2\lambda {\left\|\omega \right\|}^{2}$. T is the number of leaf nodes, *γ* is the weight of leaf nodes, and *λ* and *ω* are regular coefficients.

Combining Formulas (1), (2) and Taylor expansion of the loss function, Formula (3) is obtained as follows:

(3) $$\begin{array}{l} L^{\left(t\right)}=\sum_{i=1}^{n}l[y_{i},{\overset{\wedge }{y}}_{i}^{\left(t-1\right)}+f_{i}(x_{i})]+\Omega (f_{t})+\sum_{k=1}^{t-1}\Omega (f_{k})=\\ \sum_{i=1}^{n}\left[l(y_{i},{\overset{\wedge }{y}}_{i}^{\left(t-1\right)})+g_{i}f_{t}(x_{i})+1/2h_{i}f_{t}^{2}(x_{i})\right]+\Omega (f_{t})+\sum_{k=1}^{t-1}\Omega (f_{k})=\\ \sum_{i=1}^{n}\left[g_{i}f_{t}(x_{i})+1/2h_{i}f_{t}^{2}(x_{i})\right]+\gamma T+1/2\lambda \omega_{j}^{2}+C \end{array}$$

$g_{i}$ is the first derivative, $h_{i}$ is the second derivative, and *C* is a constant. The formula is as follows:

(4) $$g_{i}=\partial_{{\overset{\wedge }{y}}_{i}^{\left[t-1\right]}}l(y_{i},{\overset{\wedge }{y}}_{i}^{t-1}),$$

(5) $$h_{i}=\partial_{{\overset{\wedge }{y}}_{i}^{\left[t-1\right]}}^{2}l(y_{i},{\overset{\wedge }{y}}_{i}^{t-1}),$$

(6) $$C=\sum_{i}^{n}l(y_{i},y_{i}^{\left[t-1\right]})+\sum_{k=1}^{t-1}\Omega (f_{k}),$$

Definition $I_{j}=\{i|q(x_{i})=j\}$ represents a sample set of leaf node *j*. After removing the constant term from Formula (3), the derivative term is 0, and the optimal solution $\omega_{j}^{*}$ can be obtained as follows:

(7) $$\omega_{j}^{*}=-\frac{G_{j}}{H_{j}+\lambda },$$

(8) $$G_{j}=\sum_{i\in I_{j}}g_{i},$$

(9) $$H_{j}=\sum_{i\in I_{j}}h_{i},$$

After bringing the optimal solution $\omega_{j}^{*}$ into Formula (3), we get Formula (10):

(10) $$L^{\left(t\right)}=-1/2\sum_{j=1}^{T}\frac{G_{j}^{2}}{H_{j}+\lambda }+\gamma T+C,$$

XGBoost uses the greedy algorithm to segment the existing nodes each time. Assuming that IL and IR are the set of left and right nodes after segmentation, I = IL∪IR, then the information gain after segmentation is:

(11) $$L_{\left(split\right)}=Gain=1/2[\frac{G_{L}^{2}}{H_{L}+\lambda }+\frac{G_{R}^{2}}{H_{R}+\lambda }+\frac{{\left(G_{L}+G_{R}\right)}^{2}}{H_{L}+H_{R}+\lambda }]-\gamma ,$$

(12) $$G_{L}=\sum_{i\in I_{L}}g_{i},G_{R}=\sum_{i\in I_{R}}g_{i},H_{L}=\sum_{i\in I_{L}}h_{i},H_{R}=\sum_{i\in I_{R}}h_{i}$$

As can be seen from Formula (11), similar to the ID3, C4.5, and CART decision tree algorithms, XGBoost determines whether a node is being split by subtracting the unsplit node score from the split left and right node scores. Meanwhile, XGBoost considers the complexity of the model and adds a regular term λ to limit the growth of the tree. When the gain is less than λ, no node splitting is performed.

#### 2.3.2. XGBoost Hypertension Outcomes Prediction Process

**Figure 5:**
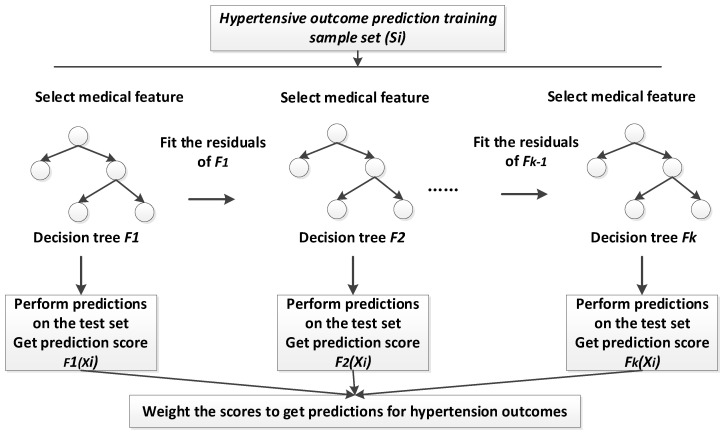
XGBoost-based Hypertensive Outcomes Prediction Modeling Process.

The XGBoost algorithm is used to establish a model for prediction of hypertension outcomes. Figure 5 is the process of modeling of XGBoost.

**Figure 6:**
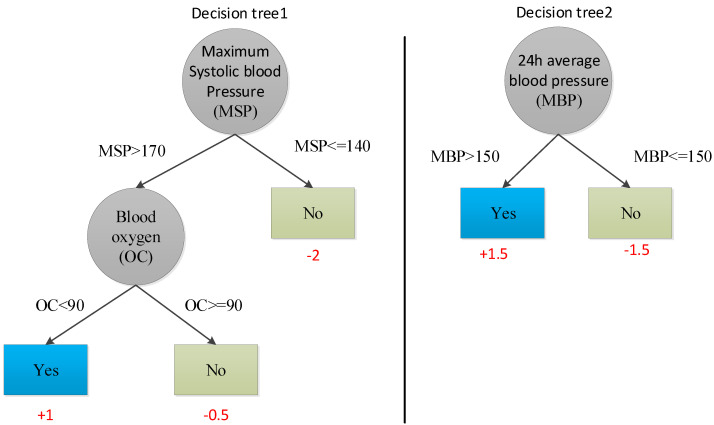
Example of XGBoost-based prediction model.

Figure 6 is an example of modeling using XGBoost. Two decision trees are generated based on the two characteristics of maximum systolic blood pressure and 24-h average blood pressure. For determining whether an outcome occurs in a sample, there are corresponding scores on the leaf nodes of the two trees. The scores of the two trees are summed up, the score of “outcome occurrence” is 2.5, and the score of “no outcome occurrence” is 4, so it is judged that the sample would not have an outcome.

### 2.4. Analysis and Optimization of GSFTS-FS and XGBoost Parameters

The number of parameters to be determined in this paper is small and the value range is relatively easy to determine. Therefore, the grid search with cross validation is selected as the parameter optimization method with F1 as the evaluation index. The following parameters need to be adjusted and optimized.

(1) Candidate Set Length (CSL): the larger the length of the candidate set, the more feasible solutions can be selected in the neighborhood, and the easier it is to find the global optimal solution. However, if the length is too long, the amount of calculation will be large, and if the length is too short, it will easily fall into the local optimal solution.

(2) Tabu list length (TLL): the smaller the TLL, the larger the search range, but it is easy to repeat the search. If the TLL is too long, the calculation time will become longer.

(3) Number of iterations: the more iterations, the easier it is to find a better solution. When it reaches a certain number (saturation point), the effect will not fluctuate greatly.

(4) Max depth of the tree in XGBoost: it is used to avoid overfitting. The larger the value, the more specific samples the model will learn.

(5) Number of estimators in XGBoost (NE): the more classifiers, the better the performance of the ensemble learning model. However, too many base classifiers will not only make it more computationally expensive and slower, but also cause overfitting.

### 2.5. Hypertension Outcomes Prediction Model Based on GSFTS-FS and XGBoost

**Figure 7:**
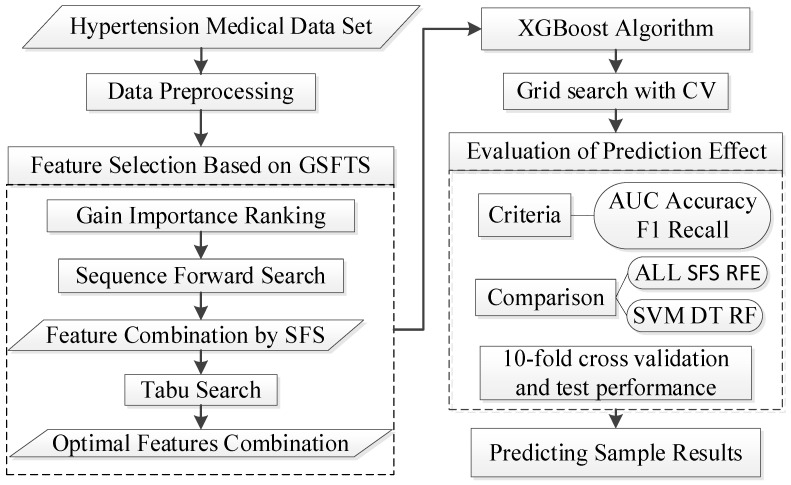
Outcome prediction model for hypertension patients.

Combining the GSFTS-FS and XGBoost methods mentioned above, the hypertension outcomes prediction model can be established. The prediction model flowchart is shown in Figure 7. After comparison and verification, the optimal feature combination is determined and used as the input of the final XGBoost prediction model for clinical practical application in patients with hypertension.

## 3. Results

### 3.1. Preprocessed Medical Data

**Figure 8:**
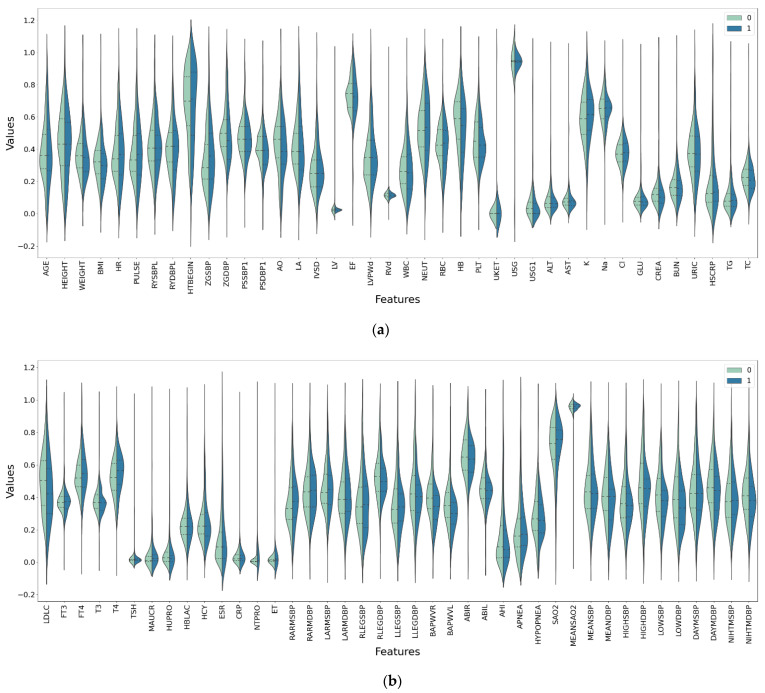
Violin plot of medical data after preprocessing. (**a**) The first 40 features. (**b**) The last 41 features.

**Figure 9:**
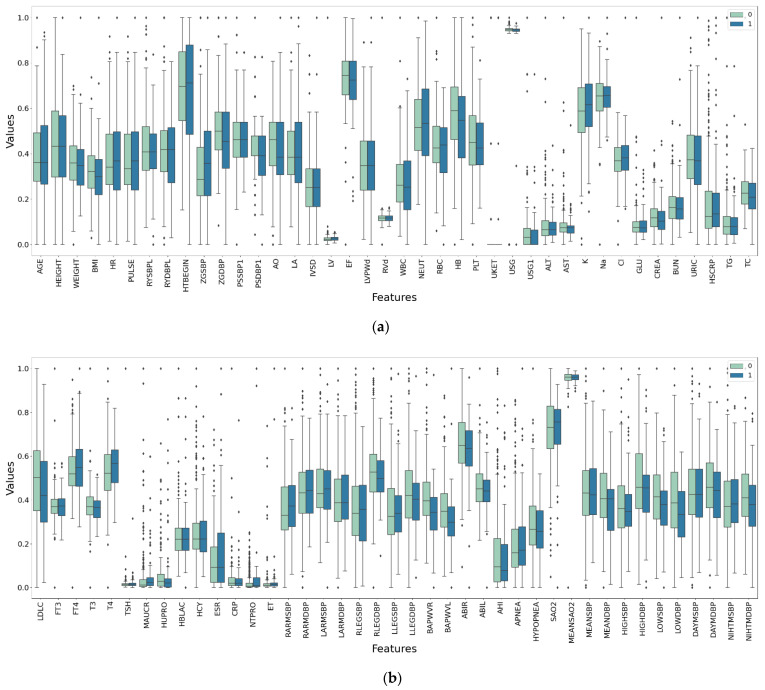
Box plot of medical data after preprocessing. (**a**) The first 40 features. (**b**) The last 41 features.

**Figure 10:**
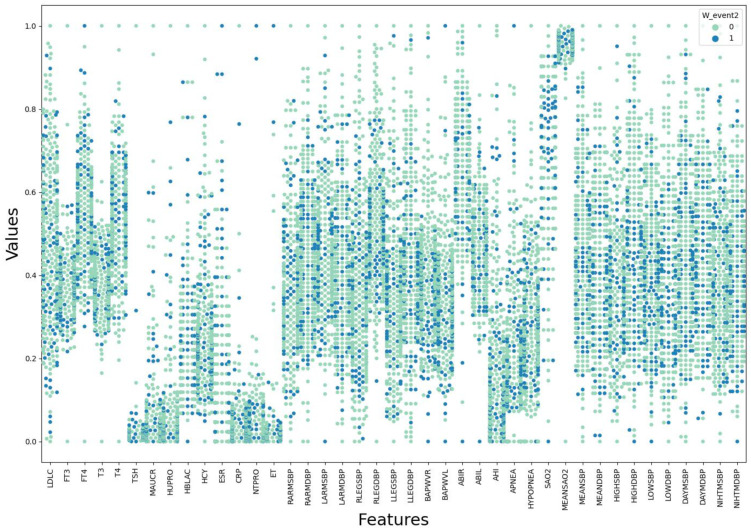
Clustered scatter plot of medical data after preprocessing.

The violin plot, box plot, and clustered scatter plot of the preprocessed data are shown in Figure 8, Figure 9 and Figure 10, respectively. Moreover, 0 means that hypertension outcomes did not occur, 1 means that hypertension outcomes occurred.

Through missing value processing, the number of samples in the data set is 752; the feature dimension is 84. The ratio of positive and negative samples is 1:7, which belongs to the category imbalanced data set. This paper adopts the EasyEnsemble method to deal with the imbalance of categories.

### 3.2. Feature Selection Results Based on GSFTS-FS

**Figure 11:**
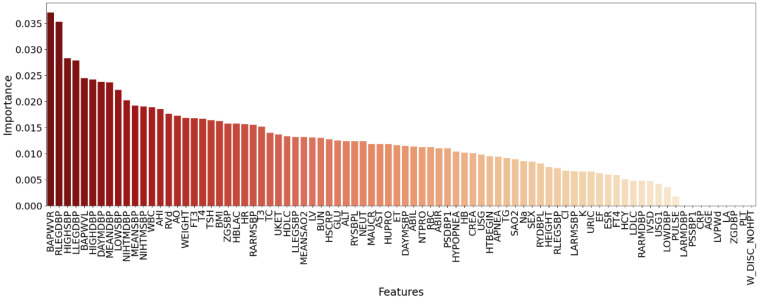
Feature importance ranking graph based on XGBoost.

The gain sequence forward tabu search feature selection (GSFTS-FS) proposed in this study is performed and has achieved some good results. Feature gain importance ranking based on XGBoost are shown in Figure 11. The feature combinations after SFS under the four evaluation criteria are obtained, as shown in Table 3. It shows the feature combinations obtained by SFS under different evaluation standards are not exactly the same.

The length of the candidate set, the length of the tabu list, and the number of iterations in GSFTS-FS need to be further adjusted and optimized before it is used to optimize the feature combination selected by SFS.

**Figure 12:**
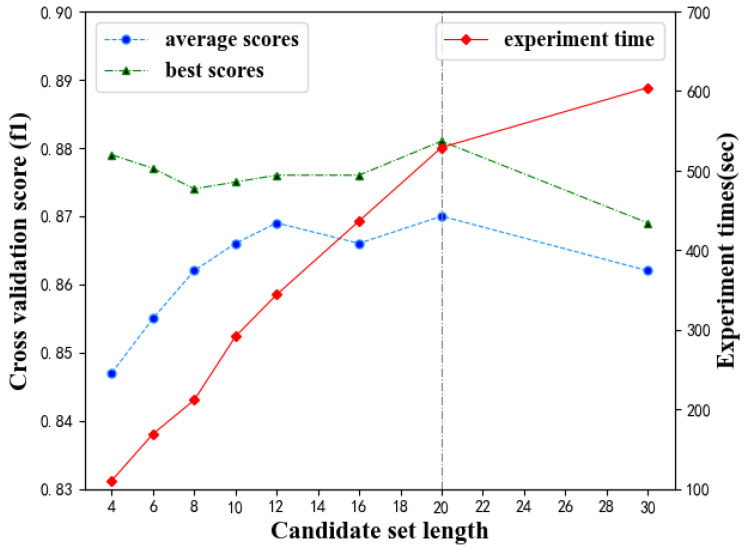
Effect of CSL on prediction model performance.

The candidate set length (CSL) is optimized first. The tabu list length is fixed at 2 and the number of iterations is fixed at 80. The performance of the prediction model under different CSL is obtained, as shown in Table 4 and Figure 12. Table 4 and Figure 12 show that the length of the candidate set has an impact on the prediction model. Both the best F1 value and the average F1 value in 80 iterations change with CSL. The optimal CSL is 20, with which the prediction model performs best. Besides, as CSL increases, the model computation time increases significantly. This means that the increase in CSL will obviously bring more computation.

**Figure 13:**
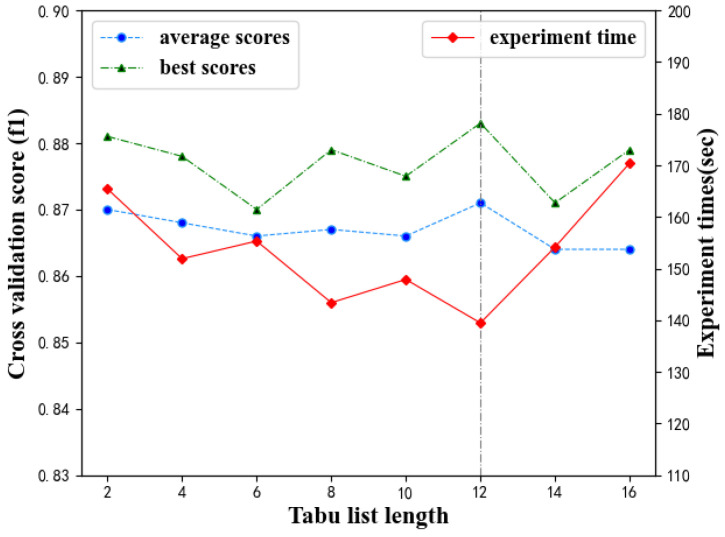
Effect of TLL on prediction model performance.

For the optimal CSL, the performance of the prediction model under different tabu list length (TLL) is shown in Table 5 and Figure 13. Table 5 shows that when the TLL reaches 12, the model performs best. Therefore, the optimal value is 12. Figure 13 shows that the performance and the calculation time does not change significantly with the increase TLL. This parameter has a small impact on the model.

**Figure 14:**
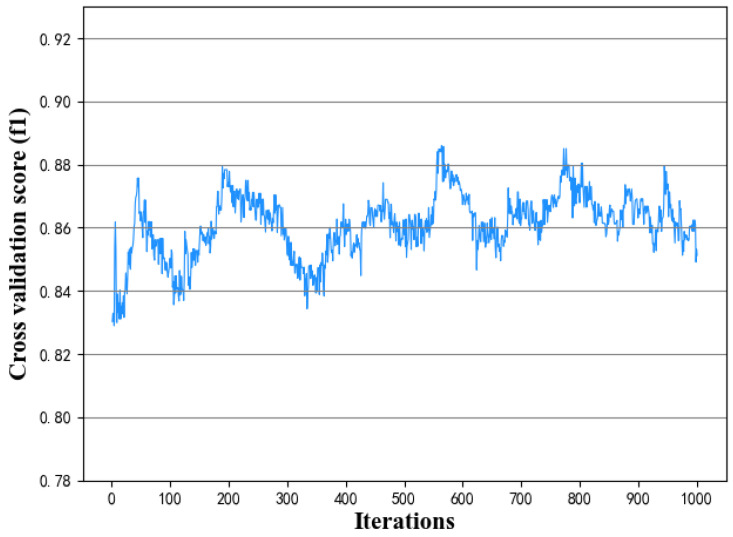
Effect of the iterations on prediction model performance.

For the optimal CSL and TLL, the model performance with increasing iterations is shown in Figure 14. It shows that with the increase of the number of iterations, the performance fluctuates up and down, but the fluctuation range of F1 value is about [0.84, 0.88] around 0.86, with no significant change. When the number of iterations is around 200, the model performs well. Excessive iterations do not improve model significantly. Besides, the more iterations, the longer the computation time. Therefore, the optimal number of iterations of the GSFTS-FS model is 200.

Now, all GSFTS-FS parameters have been determined. The tabu search is now run with the feature combinations in Table 3 as the initial solution to obtain the final optimized feature combinations for the prediction model. The results are shown in Table 6. The number of features in the feature combination by GSFTS-FS is 9–16, which is relatively small compared to the original 84 features.

### 3.3. Verification and Evaluation of Hypertension Outcomes Prediction Model

#### 3.3.1. XGBoost Parameter Tuning

**Figure 15:**
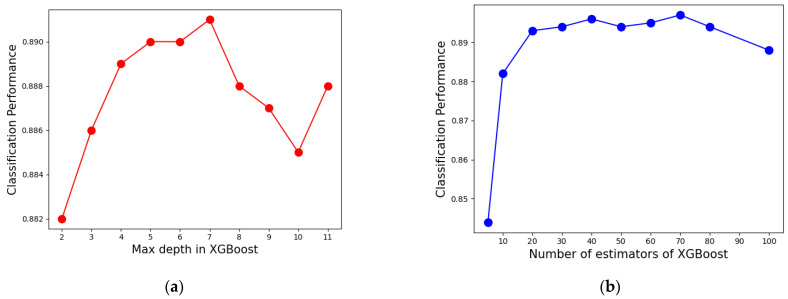
Effect of parameters on XGBoost: (**a**) max depth; (**b**) number of estimators.

The results of parameter grid search tuning of XGBoost are shown in Table 7. Therefore, the maximum tree depth and the number of trees are determined to be 7 and 70 respectively. The trends of XGBoost performance with increasing max depth and NE are shown in Figure 15.

#### 3.3.2. Prediction Model Validation Results

The hypertension outcomes prediction model proposed in this paper is verified below. The verification methods included 10-fold cross-validation and test set evaluation. We divided the medical data into training set, validation set, and test set. The training set and the validation set account for 75% of the data volume and are used for 10-fold cross training and validation. The test set accounts for 25% of the data volume and is completely independent from the model optimization procedure. The evaluation results of the test set can show the generalization performance of the model.

In order to verify the superiority of the proposed method, we compared the existing different feature selection methods and classification methods. For feature selection, the input data are the complete feature set and three feature combinations obtained by SFS, recursive feature elimination (RFE) and GSFTS. For outcome prediction, the support vector machine (SVM), decision tree (DT) and random forest (RF) are used for comparison. The model performance evaluation criteria are accuracy, AUC, F1, and recall. The results are shown in Table 8 and Table 9.

The McNemar statistical test is to verify the results in Table 9. At the significance level α = 0.05, we used McNemar statistic $\tau_{\chi^{2}}$ and the corresponding P-value to provide the quantitative information for the significance of the difference between methods. The significance analysis results are shown in Table 10, Table 11 for the feature selection methods and are shown in Table 12, and Table 13 for the prediction algorithms.

**Figure 16:**
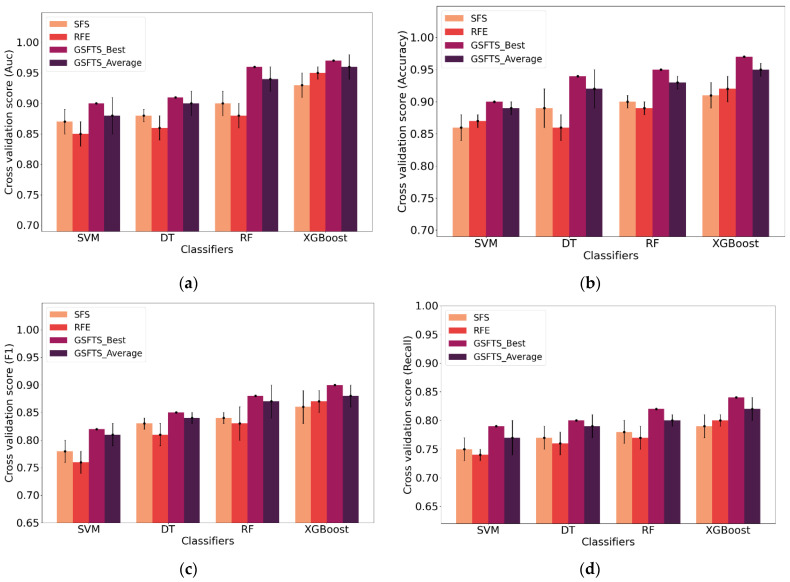
Performance comparison of the four prediction models under the three sets of feature combination: (**a**) AUC; (**b**) accuracy; (**c**) F1; (**d**) recall.

Figure 16 is the performance comparison of the four prediction models under the three sets of feature combination. We compare the average and optimal values in the 80 iterations of the GSFTS-FS algorithm with the values of SFS and RFE.

## 4. Discussion

Figure 8, Figure 9 and Figure 10 show the distribution of all medical data on different indicators. It can be seen from the figures that the data distribution on some indicators is the same for patients with outcomes and those without outcomes, while the data distribution on other indicators is different for different patients. This shows that some medical characteristics can affect hypertension outcomes, resulting in differences in data distribution.

Feature combinations by GSFTS-FS in Table 6 show that the number of medical indicators used to predict hypertension outcomes has been greatly reduced compared with the original medical indicators set. For doctors, this helps them narrow the scope of analysis and analyze pathologically the factors that affect hypertension outcomes. For example, the No. 61 index (right brachial-ankle pulse wave conduction velocity) is required by the prediction model under all evaluation indices in Table 6. For patients, fewer medical indicators mean fewer examination items. For machine learning algorithms, the optimal combination of features can reduce learning burden and calculation time, to better judge the hypertension outcomes with high efficiency.

Now, we can analyze the impact of different feature combinations on the prediction model from Table 8. No matter what kind of evaluation index, the prediction result of the feature combination obtained by GSFTS-FS is significantly better than that without feature selection. The GSFTS-FS method significantly improves the performance of the prediction model. The feature combinations obtained by SFS and RFE also help to improve performance, but it is not as good as the feature combination optimized by GSFTS-FS, which has a higher promotion rate.

Next, we analyze the performance of the four prediction models. It can be seen from Table 8 that the performance of the XGBoost is better than SVM, C4.5 decision tree, or random forest under any evaluation criteria, and the advantage is obvious. The XGBoost classification algorithm combined with the GSFTS-FS algorithm performs the best, with an accuracy of 0.95, an AUC value of 0.96, an F1 value of 0.88, and a recall value of 0.82. Compared with the dataset without feature selection, the accuracy, AUC value, F1 value, and recall value of this method have been increased by 7.9%, 7.9%, 8.6%, and 15.5%, respectively.

Table 9 shows that the prediction has good generalization ability and can be used for the prediction of new samples. The GSFTS-XGB achieved the best performance on the test set and its AUC, accuracy, F1 value, recall are 0.92, 0.94, 0.87, 0.80, respectively. The results of McNemar’s significance analysis confirmed that, the proposed methods is superior to alternative approaches. The results in Table 10 and Table 11 show that new medical feature selection method GSFTS has significant advantages over other existing methods ($\tau_{\chi^{2}}$ > 3.8415 or *p*-value < 0.05) while there is no significant difference between RFE and SFS. As shown in Table 12 and Table 13, the difference between XGBoost and other classification algorithms are significant within 95% confidence interval.

By observing Figure 16, the same conclusions as in Table 8 can be obtained. The GSFTS-FS method is more effective than SFS method, and the performance is significantly improved. The XGBoost performed better than the other two classification algorithms.

Compared with similar work [17,18,19,20,21,22], we not only proved that XGBoost is better than random forest and decision tree that are commonly used in the literature in predicting hypertension diseases, but also further optimized the selection process of medical features to better quantitatively analyze which medical indicators affect hypertension outcomes. For example, Liu Y used a combination of recursive feature elimination (RFE) and XGBoost to build a hypertension prediction model [16]. RFE is a greedy optimization algorithm, which is easy to fall into a local optimal solution, while GSFTS-FS can solve this problem and obtain a better global optimal solution.

The study in this paper has the following points for further research. First, due to the difficulty in collecting medical data, the amount of data used in this research is still small. With more samples accumulated and collected in the future, the model can be further optimized and adjusted. Second, the Recall value of the prediction model established in this paper is about 80%. For clinical applications, it is hoped that patients who are about to have an outcome will be predicted as many as possible. Due to the characteristics of hypertension, the incidence of outcomes is small, and further research is needed to improve the Recall value of the prediction model.

## 5. Conclusions

One difficult question in medicine is, “Will serious outcomes occur in patients with hypertension?”

Research derived from clinical medicine has not solved this question. In regards to data mining and machine learning, we proposed a hypertensive outcome prediction model combining GSFTS-FS and XGBoost. We analyzed the medical data of 1357 patients with hypertension from a Beijing hospital, and verified the prediction model. By comparing and analyzing the experimental results, we can draw the following conclusions: (1) GSFTS-FS can screen valuable parts from many medical indicators and provide high-quality input variables for the prediction model. (2) GSFTS-FS captures the changes in the information gain of medical variables and digs out key factors affecting the hypertension outcomes through global optimal search. (3) Through GSFTS-FS, we discovered that medical variables, such as right brachial-ankle pulse wave conduction velocity, the highest systolic blood pressure, limb blood pressure, and ambulatory blood pressure may have a higher impact on hypertension outcomes. (4) The prediction model combining the GSFTS-FS method and XGBoost algorithm performs well and has an accuracy of 0.946, an AUC of 0.956, an F1 of 0.879, and a recall of 0.805, which can accurately and effectively predict outcomes in patients with hypertension.

The model proposed in this paper can provide guidance and aid decision-making for doctors in clinical diagnosis and treatment, and has the significance of theoretical research and practical application. First, the model greatly reduces the number of medical indicators needed to determine the occurrence of outcome. Instead of a full set of inspections, patients only need to do targeted inspection items. Second, the model can accurately determine whether a patient will have a hypertension outcome. If the patient is predicted to have an outcome, the doctor can prepare in advance and take protective measures. This can help reduce the incidence of outcomes or enable patients to be treated promptly and properly when outcomes occur. Third, the model can indicate which medical indicators have an impact on the outcome. It provides guidance for medical research. Experts and scholars in the medical field can analyze the correlation between various indicators and hypertension outcomes from a pathological perspective.

## Figures and Tables

**Table 1:** Description of partial hypertension examination indicators.

No.	Name	Description	Type	Value Range	Mean Value	Std.
1	Sex	baseline data	Categorical	Male or Female (1 or 0)	/	/
2	AGE	baseline data	Numeric	15–76	38.31	11.42
3	BMI	body mass index	Numeric	16.32–50.93	27.35	4.19
4	HR	heart rate	Numeric	49–121	76.28	12.71
5	RYDBPL	left arm diastolic pressure	Numeric	57–160	98.53	16.8
6	EF	ejection fraction	Numeric	30–77	64.34	5.81
7	NEUT	percentage of neutrophils	Numeric	36.1–82.6	60.77	7.89
8	FT4	free thyroxine	Numeric	0.32–1.91	1.17	0.188
9	MAUCR	urinary microalbumin/creatinine	Numeric	0–1081.26	59.56	133.67
10	ESR	erythrocyte sedimentation rate	Numeric	1–44	7.21	6.89
11	ET	endothelin	Numeric	0.1–8.47	0.32	0.76
12	RARMSBP	right upper limb systolic blood pressure	Numeric	100–233	149.05	20.91
13	BAPWVR	right brachial-ankle pulse wave conduction velocity	Numeric	7.3–28.6	15.65	3.05
14	HIGHSBP	the highest systolic blood pressure	Numeric	152.25–242	166.17	20.13

**Table 2:** Tabu list example.

Tabu List (*TL* = 3)	
NO.	Tabu target
1	F2
2	
3	

**Table 3:** Feature combination by SFS.

Criteria	Feature Combinations
AUC	61, 64, 70, 63, 65, 75, 71, 69, 72, 77, 68, 76, 22, 78, 21, 15, 4, 44, 47, 48, 5, 11, 51, 6, 56, 46, 41, 27, 42, 60, 82, 18, 37, 39, 35, 30, 8, 23
ACC	61, 64, 70, 63, 65, 75, 71, 69, 72, 77
F1	61, 64, 70, 63, 65, 75, 71, 69, 72, 77, 68
Recall	61, 64, 70, 63, 65, 75, 71, 69

**Table 4:** Performance of prediction model under different CSL.

CSL Criterion	4	6	8	10	12	16	20	30
F1-average	0.847	0.855	0.862	0.866	0.869	0.866	0.870	0.862
F1-best	0.879	0.877	0.874	0.875	0.876	0.876	0.881	0.869
Time(s)	110.55	169.21	211.71	291.63	344.18	436.79	529.14	604.32

**Table 5:** Performance of prediction model under different TLL.

TLL Criterion	2	4	6	8	10	12	14	16
F1-average	0.870	0.868	0.866	0.867	0.866	0.871	0.864	0.864
F1-best	0.881	0.878	0.870	0.879	0.875	0.883	0.871	0.879
Time(s)	165.41	151.90	155.32	143.40	147.89	139.47	154.15	170.40

**Table 6:** Feature combination by GSFTS-FS.

Criteria	Feature Combinations
ACC	61, 70, 63, 75, 72, 77, 68, 76, 21, 15, 51, 27, 42, 35, 8, 23
AUC	61, 70, 63, 75, 71, 72, 68, 15, 5
F1	61, 70, 63, 32, 72, 76, 15, 4, 47, 60
Recall	61, 70, 63, 51, 21, 77, 68, 15, 41

**Table 7:** XGBoost performance under different parameters.

Max_Depth	f1	NE	f1
2	0.882	5	0.844
3	0.886	10	0.880
4	0.889	20	0.893
5	0.890	30	0.894
6	0.890	40	0.896
7	0.891	50	0.894
8	0.888	60	0.895
9	0.887	70	0.897
10	0.885	80	0.894
11	0.888	100	0.888

**Table 8:** Prediction results of different methods and criterion using 10-fold cross validation.

Criterion	AUC				Accuracy			
Model Feature Combination	SVM	DT	RF	XGB	SVM	DT	RF	XGB
ALL	0.71 ± 0.03	0.75 ± 0.05	0.78 ± 0.04	0.89 ± 0.05	0.76 ± 0.03	0.78 ± 0.02	0.79 ± 0.04	0.88 ± 0.03
SFS	0.87 ± 0.02	0.88 ± 0.01	0.90 ± 0.02	0.93 ± 0.02	0.86 ± 0.02	0.89 ± 0.03	0.90 ± 0.01	0.91 ± 0.02
(Increase rate)	22.5%	17.3%	17.9%	4.5%	13.1%	14.1%	13.9%	3.4%
RFE	0.85 ± 0.02	0.86 ± 0.02	0.88 ± 0.02	0.95 ± 0.01	0.87 ± 0.01	0.86 ± 0.02	0.89 ± 0.01	0.92 ± 0.02
(Increase rate)	19.7%	14.7%	11.5%	6.7%	14.5%	10.3%	12.7%	4.5%
GSFTS-FS	0.88 ± 0.03	0.90 ± 0.02	0.94 ± 0.02	0.96 ± 0.02	0.89 ± 0.01	0.92 ± 0.03	0.93 ± 0.01	0.95 ± 0.01
(Increase rate)	23.9%	20.0%	20.5%	7.9%	17.1%	17.9%	17.7%	7.9%
Criterion		F1				Recall		
Model Feature Combination	SVM	DT	RF	XGB	SVM	DT	RF	XGB
ALL	0.71 ± 0.03	0.72 ± 0.04	0.62 ± 0.02	0.81 ± 0.03	0.65 ± 0.02	0.67 ± 0.03	0.66 ± 0.02	0.71 ± 0.02
SFS	0.78 ± 0.02	0.83 ± 0.01	0.84 ± 0.01	0.86 ± 0.03	0.75 ± 0.02	0.77 ± 0.02	0.78 ± 0.02	0.79 ± 0.02
(Increase rate)	9.9%	15.3%	35.5%	6.2%	15.3%	14.9%	12.8%	11.3%
RFE	0.76 ± 0.02	0.81 ± 0.02	0.83 ± 0.03	0.87 ± 0.02	0.74 ± 0.01	0.76 ± 0.02	0.77 ± 0.02	0.80 ± 0.01
(Increase rate)	7.0%	12.5%	33.8%	7.4%	13.9%	13.4%	16.7%	12.7%
GSFTS-FS	0.81 ± 0.02	0.84 ± 0.01	0.87 ± 0.03	0.88 ± 0.02	0.77 ± 0.03	0.79 ± 0.02	0.80 ± 0.01	0.82 ± 0.02
(Increase rate)	14.1%	16.6%	40.3%	8.6%	18.5%	17.9%	21.2%	15.5%

**Table 9:** Prediction results of different methods and criterion on test set.

Criterion	AUC				Accuracy			
Model Feature Combination	SVM	DT	RF	XGB	SVM	DT	RF	XGB
ALL	0.70	0.75	0.76	0.87	0.68	0.70	0.72	0.80
SFS	0.83	0.86	0.87	0.89	0.75	0.77	0.80	0.85
(Increase rate)	18.5%	14.7%	14.5%	2.3%	10.3%	10.0%	11.1%	6.3%
RFE	0.82	0.85	0.88	0.90	0.74	0.80	0.83	0.84
(Increase rate)	17.1%	13.3%	15.8%	3.4%	8.8%	14.2%	15.2%	5.0%
GSFTS-FS	0.87	0.87	0.90	0.92	0.76	0.82	0.88	0.94
(Increase rate)	24.2%	16.0%	18.4%	5.7%	11.8%	17.1%	22.2%	17.5%
Criterion		F1				Recall		
Model Feature Combination	SVM	DT	RF	XGB	SVM	DT	RF	XGB
ALL	0.68	0.69	0.61	0.79	0.61	0.62	0.65	0.68
SFS	0.74	0.79	0.82	0.83	0.70	0.71	0.72	0.76
(Increase rate)	8.8%	14.5%	34.4%	5.1%	14.8%	14.5%	10.8%	11.8%
RFE	0.73	0.74	0.84	0.85	0.68	0.69	0.74	0.79
(Increase rate)	7.4%	7.2%	37.7%	7.6%	11.5%	11.3%	13.8%	16.2%
GSFTS-FS	0.78	0.79	0.85	0.87	0.72	0.73	0.78	0.80
(Increase rate)	14.7%	14.5%	39.3%	10.1%	18.0%	17.7%	20%	17.6%

**Table 10:** McNemar statistic matrix for different feature selection methods.

	SFS	RFE	GSFTS
SFS	/	0.045	4.050
RFE	0.045	/	4.762
GSFTS	4.050	4.762	/

**Table 11:** P-value matrix for different feature selection methods.

	SFS	RFE	GSFTS
SFS	/	0.831	0.044
RFE	0.831	/	0.029
GSFTS	0.044	0.029	/

**Table 12:** McNemar statistic matrix for different prediction algorithms.

	SVM	DT	RF	XGBoost
SVM	/	4.167	8.643	15.429
DT	4.167	/	4.161	8.471
RF	8.643	4.161	/	4.000
XGBoost	15.429	8.471	4.000	/

**Table 13:** P-value matrix for different prediction algorithms.

	SVM	DT	RF	XGBoost
SVM	/	0.041	0.003	0.001
DT	0.041	/	0.047	0.004
RF	0.003	0.047	/	0.046
XGBoost	0.001	0.004	0.046	/

## Data Availability

The data presented in this study are available on request from the corresponding author with required ethical review. The data are not publicly available due to ethical review.

## Appendix A

Table A1 is a list of all the medical features that are considered in this study.

**Table A1:** Medical Features.

No.	Name	Explanation
	Baseline Data	
1	SEX	sex
2	AGE	age
3	HEIGHT	height
4	WEIGHT	weight
5	BMI	body mass index
6	HR	heart rate
7	PULSE	pulse
8	RYSBPL	left arm systolic pressure
9	RYDBPL	left arm diastolic pressure
10	HTBEGIN	initial hypertension age
11	ZGSBP	highest systolic blood pressure
12	ZGDBP	highest diastolic blood pressure
13	PSSBP1	normal systolic blood pressure
14	PSDBP1	normal diastolic blood pressure
	UCG cardiac vascular ultrasound	
15	AO	ascending aorta diameter
16	LA	left atrium
17	IVSD	ventricular septal thickness
18	LV	left ventricular end diastolic diameter
19	EF	ejection fraction
20	LVPWd	thickness of the back wall
21	RVd	right ventricle
	blood routine	
22	WBC	white blood cell
23	NEUT	percentage of neutrophils
24	RBC	red blood cells
25	HB	hemoglobin
26	PLT	platelet
	Urine routine	
27	UKET	ketone body
28	USG	specific gravity of urine
29	USG1	USG tube type
	Blood biochemical	
30	ALT	alanine aminotransferase
31	AST	aspartate aminotransferase
32	K	serum potassium
33	Na	serum sodium
34	Cl	serum chlorine
35	GLU	blood sugar
36	CREA	creatinine
37	BUN	urea nitrogen
38	URIC	uric acid
39	HSCRP	high-sensitivity C-reactive protein
40	TG	triglyceride
41	TC	triacylglycerol
42	HDLC	high density lipoprotein cholesterol
43	LDLC	low density lipoprotein cholesterol
	Thyroid function	
44	FT3	serum free triiodothyronine
45	FT4	free thyroxine
46	T3	triiodothyronine
47	T4	tetraiodothyronine
48	TSH	thyroid stimulating hormone
	Urine protein	
49	MAUCR	urinary microalbumin/creatinine
50	HUPRO	4-h urine protein quantitation
	Blood sugar	
51	HBLAC	glycated hemoglobin
	Inflammatory factor	
52	ESR	erythrocyte sedimentation rate
53	CRP	C-reactive protein
54	NTPRO	amino terminal precursor protein of brain natural peptide
55	ET	endothelin
	Limb blood pressure	
56	RARMSBP	right upper limb systolic blood pressure
57	RARMDBP	right upper limb diastolic blood pressure
58	LARMSBP	left upper limb systolic blood pressure
59	LARMDBP	left upper limb diastolic blood pressure
60	LLEGSBP	left lower extremity systolic blood pressure
61	BAPWVR	right brachial-ankle pulse wave conduction velocity
62	RLEGSBP	right lower limb systolic blood pressure
63	LLEGDBP	left lower extremity diastolic blood pressure
64	RLEGDBP	right lower limb diastolic blood pressure
65	BAPWVL	left brachial-ankle pulse wave conduction velocity
66	ABIR	right ankle-brachium index
67	ABIL	left ankle-brachium index
	Dynamic blood pressure	
68	MEANSBP	24 h mean systolic blood pressure
69	MEANDBP	24 h mean diastolic blood pressure
70	HIGHSBP	the highest systolic blood pressure
71	DAYMDBP	daytime mean diastolic blood pressure
72	LOWSBP	the lowest systolic blood pressure
73	LOWDBP	the lowest diastolic blood pressure
74	DAYMSBP	daytime average systolic blood pressure
75	HIGHDBP	the highest diastolic blood pressure
76	NIHTMSBP	nighttime average systolic blood pressure
77	NIHTMDBP	nighttime average diastolic blood pressure
	Breathing sleep	
78	AHI	hourly breathing number
79	APNEA	the longest apnea number
80	HYPOPNEA	the longest hypoventilation time
81	SAO2	the lowest SaO2%
82	MEANSAO2	the average SaO2%
	Other	
83	HCY	homocysteine
84	W_DISC_NOHPT	number of antihypertensive drugs at discharge
